# Analysis of Cellular Damage Resulting from Exposure of Bacteria to Graphene Oxide and Hybrids Using Fourier Transform Infrared Spectroscopy

**DOI:** 10.3390/antibiotics12040776

**Published:** 2023-04-18

**Authors:** Christopher M. Liauw, Misha Vaidya, Anthony J. Slate, Niall A. Hickey, Steven Ryder, Emiliano Martínez-Periñán, Andrew J. McBain, Craig E. Banks, Kathryn A. Whitehead

**Affiliations:** 1 Microbiology at Interfaces Group, School of Healthcare Sciences, Manchester Metropolitan University, Chester Street, Manchester M1 5GD, UK; chris.liauw.curvi.hifi@googlemail.com (C.M.L.);; 2 Department of Life Sciences, University of Bath, Claverton Down, Bath BA2 7AY, UK; 3 Departamento de Química Analítica y Análisis Instrumental, Universidad Autónoma de Madrid, 28049 Madrid, Spain; 4 School of Health Sciences, Faculty of Biology, Medicine and Health, The University of Manchester, Manchester M13 9PT, UK; 5 Faculty of Science and Engineering, Manchester Metropolitan University, Chester Street, Manchester M1 5GD, UK

**Keywords:** antimicrobial resistance, cellular ultrastructure, FTIR, graphene, graphene oxide hybrids, graphite

## Abstract

With the increase in antimicrobial resistance, there is an urgent need to find new antimicrobials. Four particulate antimicrobial compounds, graphite (G), graphene oxide (GO), silver–graphene oxide (Ag-GO) and zinc oxide–graphene oxide (ZnO-GO) were tested against *Enterococcus faecium*, *Escherichia coli*, *Klebsiella pneumoniae* and *Staphylococcus aureus*. The antimicrobial effects on the cellular ultrastructure were determined using Fourier transform infrared spectroscopy (FTIR), and selected FTIR spectral metrics correlated with cell damage and death arising from exposure to the GO hybrids. Ag-GO caused the most severe damage to the cellular ultrastructure, whilst GO caused intermediate damage. Graphite exposure caused unexpectedly high levels of damage to *E. coli*, whereas ZnO-GO exposure led to relatively low levels of damage. The Gram-negative bacteria demonstrated a stronger correlation between FTIR metrics, indicated by the perturbation index and the minimal bactericidal concentration (MBC). The blue shift of the combined ester carbonyl and amide I band was stronger for the Gram-negative varieties. FTIR metrics tended to provide a better assessment of cell damage based on correlation with cellular imaging and indicated that damage to the lipopolysaccharide, peptidoglycan and phospholipid bilayers had occurred. Further investigations into the cell damage caused by the GO-based materials will allow the development of this type of carbon-based multimode antimicrobials.

## 1. Introduction

Antimicrobial resistance is a global health issue which exacerbates the clinical implication of infections and can result in postsurgical complications leading to increased mortality [1,2]. This issue has been compounded by antibiotic misuse [3]. Although evidence from real-word scenarios is unclear there have been some reports that biocides and antimicrobial metals can co-select for antibiotic resistance [4,5]. There is currently a need to develop alternative antimicrobials [6], of which metal ions represent a potentially important novel class [7]. Metal ions have been used since antiquity for the treatment of infections, particularly copper (Cu) [8], gold (Au) [9] and silver (Ag) [10]. Furthermore, Cu, Au and Ag surfaces have been associated with reductions in microbial colonisation in hospital environments, which can be of use in controlling contamination and infection [11,12,13]. Other transition metals have also been investigated, with palladium (Pd) being used in surgical implants and catheters [14,15], platinum (Pt) being used in implantable devices and catheters [15,16] and zinc (Zn) being used in dental implants and surfaces [17,18]. There is also growing evidence of combinatorial effects between metals and their complexes, which could be harnessed to target multidrug-resistance bacteria [19,20]. Furthermore, complexes of multiple metals such as Ag/Cu and Ag/Pd have demonstrated a synergistic activity against antimicrobial-resistant bacteria in planktonic and biofilm forms [21]. This synergistic activity highlights the number of mechanisms of action by which metals are toxic to microorganisms and these can be combined to target resistant microorganisms. Metal-ion toxicity has been investigated over the past decade with several mechanisms suggested such as protein dysfunction, reactive oxygen species (ROS) generation, antioxidant depletion, a perturbation of the membrane, interference with nutrient uptake and genotoxicity [7].

With antimicrobial synergy identified between metal-ion solutions and non-metal antimicrobial compounds, the evaluation of antimicrobial compounds in order to target medically relevant microorganisms with multiple mechanisms of action is a high priority. Two such examples are the carbon-based compounds graphite (G) and graphene oxide (GO) [22]. Graphene is structurally composed of carbon atoms that are bonded in an sp2 (trigonal) hybridisation forming two-dimensional single atom layers. The individual layers of graphene can be bonded together by weak van der Waals interactions forming graphite. Graphene oxide is formed with the addition of oxygen-containing functional groups such as carboxylic acid, ester, ketone, phenols and epoxy. Graphite is highly utilised in the biomedical sector for drug delivery [23] among other uses [24,25]. The use of graphite as an antimicrobial is a relatively recent notion; however, some success has been found with carbon thin films which were used to reduce the adherence of *Staphylococcus aureus* and *Staphylococcus epidermidis* by 65% and 85%, respectively [26].

Many of these compounds interact with the bacterial cell envelope; however, their cellular mechanism of actions, particularly when used in synergistic combinations, are poorly understood. Gram-negative bacteria have a cell wall consisting of four distinct layers [27]. The first is an outer layer of lipopolysaccharide (LPS) that itself consists of three different regions: (i) a glycophospholipid moiety (lipid A); (ii) most of the time, lipid A moieties are linked to a core oligosaccharide via 2-keto-3-deoxy-octulosonic acid (Kdo); (iii) the third moiety of LPS is termed the O-chain and this is comprised of oligosaccharide repeating units which extend outside of the bacteria [27]. Gram-positive bacteria lack an outer membrane but have a more extensive peptidoglycan layer protecting their surface. The outer layer of a Gram-positive bacterium consists of peptidoglycan (PG) polymer chains grafted with pentapeptide stems, the latter are bridged via a range of moieties which depend on bacterial type [28,29]. The bridges effectively form cross-links between the peptide grafts on the PG chains.

With this in mind, in order to investigate the mechanisms of antimicrobial action of such compounds, clinically relevant organisms which cause hospital-associated infections with varying cellular physiology structures were selected. *Escherichia coli* is a Gram-negative bacterium, which is a part of the normal microflora of the gut [30]. In rare cases, some serotypes can be pathogenic and cause various infections, with “uropathogenic *E. coli*” (UPEC) being a major cause of 90% of urinary tract infections (UTIs) [31,32]. *Klebsiella pneumoniae* is a Gram-negative bacterium, which is predominately commensal, being a resident of the oral cavity, skin and gut [33]. *K. pneumoniae* can be an opportunist pathogen that thrives in hospital environments and is a major cause of surgical site infections and pneumonia and is second to *E. coli* in causes of UTIs [34,35]. *Enterococcus faecium* is a Gram-positive bacterium that was previously thought to be a harmless commensal microorganism of the gut flora [36]. However, *E. faecium* has become a leading cause of nosocomial infections, being an important emergent pathogen causing bacteraemia, surgical site infections and UTIs whilst also being extremely recalcitrant, being able to survive for long periods on hospital surfaces [37,38]. *S. aureus* is a Gram-positive bacterium that is predominately commensal, being a common member of the microflora of the skin, upper respiratory and gut [39,40]. However, it has serotypes that can be pathogenic and due to this, it is implicated in a wide range of infections such as abscesses, skin infections and more seriously, it is a one of the most common hospital-acquired infections and a leading cause of postoperative death [41].

In both Gram-positive and Gram-negative bacteria, there is a relatively high level of structural order, where intermolecular interactions operate from relatively fixed distances and thereby have a relatively fixed influence on the distribution of vibration frequencies of a given chemical bond. Any agent that has the effect of reducing and/or modifying the structural order will therefore give rise to shifting and broadening of the distribution of bond vibration frequencies, which can be measured by Fourier transform infrared spectroscopy (FTIR). There are also cases where the antimicrobial agent interacts with specific parts of the cell wall and perturbs the vibration frequency distribution of specific bonds. The destruction of the bacterial cell wall leads to a reduction or change in the structural order and hence resolvable changes in the infrared spectrum. The aim of this work therefore was to determine the effect of some antimicrobial compounds on the morphology and chemistry of the bacterial wall of known potential pathogens, in order to develop a greater understanding of the mode of action of such molecules.

## 2. Results

### 2.1. FTIR Spectra of Pristine Bacteria

**Figure 1:**
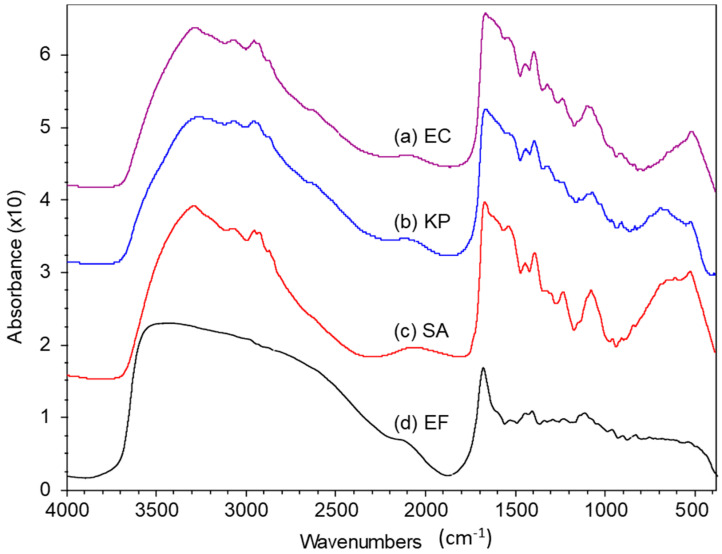
FTIR Spectra of dried bacterial call suspensions on silicon wafers; (a) *E. coli* (EC), (b) *K. pneumoniae* (KP), (c) *S. aureus* (SA) and (d) *E. faecium* (EF).

Previous work demonstrated that the amount of zinc in the compounds was determined using energy dispersive X-ray [42] to be 72.65 wt% and for silver, 69.77 wt%. The spectra of the pristine bacterial cells were determined in Figure 1. The Gram-negative *E. coli* and *K. pneumoniae* gave near-identical spectra. Interestingly, the spectrum of Gram-positive *S. aureus* was also similar to the two Gram-negatives. It was notable, that despite these latter factors, the spectrum of *E. faecium* was slightly different than those of the other three bacteria and featured an OH/NH stretching band, which peaked at 3400 cm^−1^ rather than 3300 cm^−1^, in the case of the other three bacteria. The C-H stretching vibrations included alkenic C-H, methyl asymmetric, methylene asymmetric and methyl symmetric. The C-H bonds were in the lipid bilayers, and the methyl groups were on the polysaccharide units making up the O-antigen chains and the *N*-acetyl-glucosamine-derived chains (the –HNC(O)-CH_3_ groups). The methylene groups formed the lipid bilayers and were on the pentapeptide stem of the peptidoglycan and the methyl groups of the *N*-acetyl-glucosamine.

The amide and fingerprint regions were also different. The amide I absorption was at a slightly higher frequency (1697 cm^−1^ rather than 1675–1681 cm^−1^). The C-NH_2_/C-NH_3_^+^ band (1640–1620 cm^−1^) of *E. faecium* was also of lower intensity, making the amide I peaks appear sharper. There may also have been a greater degree of confounding between the amide I and the carbonyl stretching bands of the glycerol-derived ester component of the phospholipid bilayer.

### 2.2. Effect of GO Hybrids in FTIR Spectra of Bacteria

**Figure 2:**
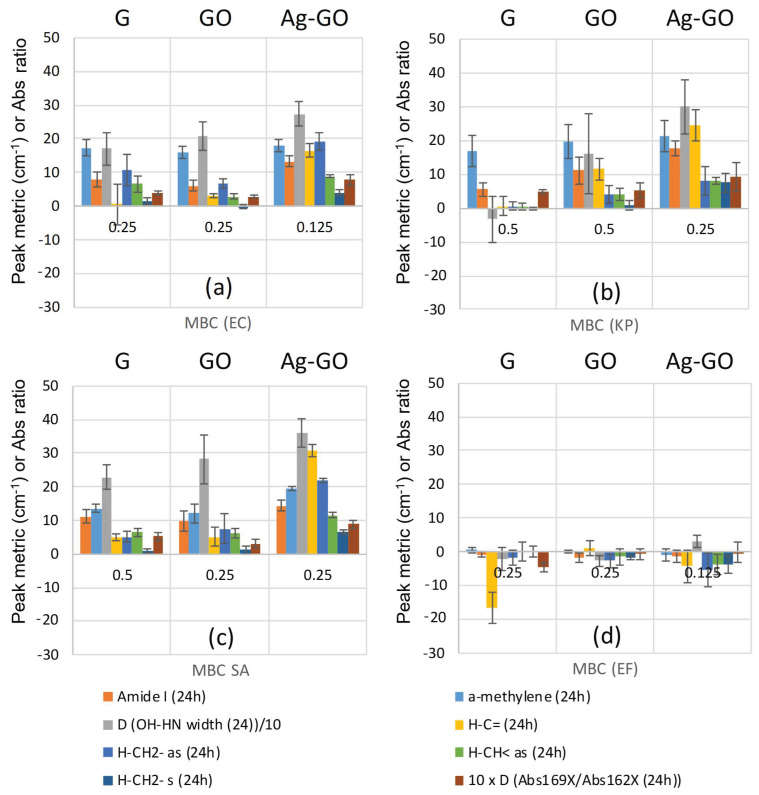
The relationship between peak metrics indicated above and minimum bactericidal concentration (MBC) for (**a**) *E. coli* (EC), (**b**) *K. pneumoniae* (KP), (**c**) *S. aureus* (SA) and (**d**) *E. faecium* (EF) (*n* = 3). The values of the disturbance index are shown at the top of each graph.

**Figure 3:**
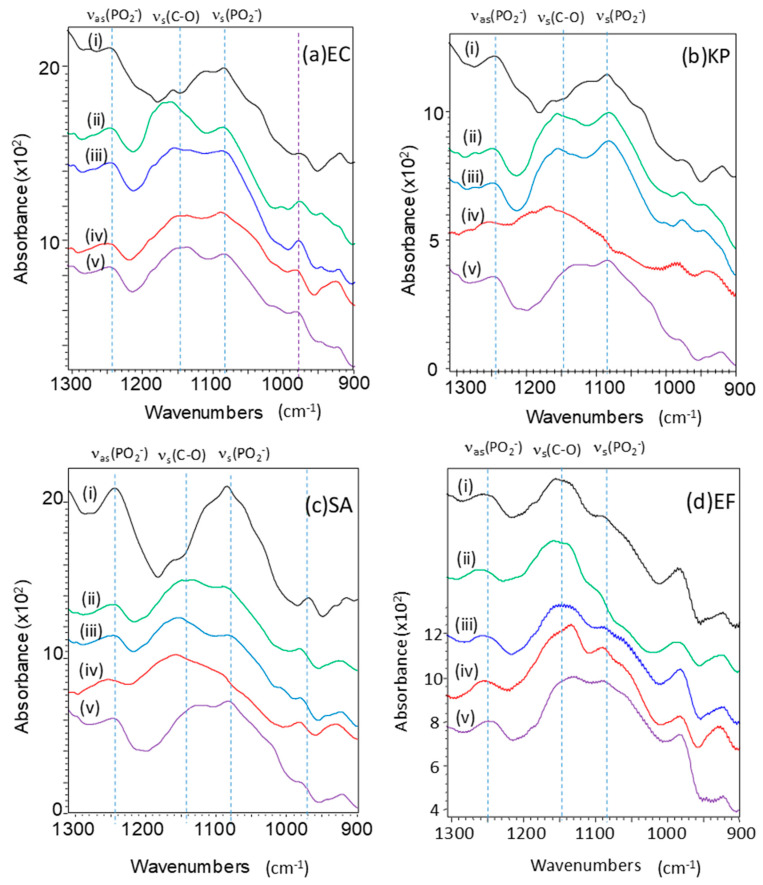
FTIR spectra showing phosphate and C-O stretching regions for (**a**) *E. coli* (EC), (**b**) *K. pneumoniae* (KP), (**c**) *S. aureus* (SA) and (**d**) *E. faecium* (EF). The individual spectra within each figure refer to the following: (i) pristine bacteria, (ii) graphite exposure, (iii) graphene oxide (GO) exposure, (iv) silver–graphene oxide exposure (Ag-GO) and (v) zinc oxide–graphene oxide exposure (ZnO-GO).

Following the treatment of the bacteria with the compounds, FTIR analyses in the diffuse reflectance cell were carried out to demonstrate the changes that occurred in the chemical spectra of the bacteria (Figure 2 and Figure 3). The principal variations included changes in the shape of the hydrogen-bonded OH and NH stretching-band envelope, shifts in various C-H stretching frequencies, the position of the amide I carbonyl stretch and the position of the absorption at ca. 1400 cm^−1^ (Figure 2).

Changes in the 4000–1800 cm^−1^ region were demonstrated (Figure 2). The OH and NH groups were due to hydroxyl groups and amide groups on the oligosaccharide repeat units of the O antigen component of the lipopolysaccharide component affixed to the outer phospholipid bilayer. N-H stretching may also be assigned to the H-N groups of the phosphatidyl-ethanolamine component of the phospholipid bilayer.

Changes in shape of the OH/NH band envelope were expressed as a change in the average peak width at half-height relative to that of the pristine bacteria. In all bacterial strains, the OH/NH peak broadened (by as much as 360 cm^−1^) as the destruction of the cellular ultrastructure progressed. The C-H stretching peaks also showed a significant shifting in all the treated bacteria; the frequencies of the alkenic, methyl asymmetric methylene and methyl symmetric stretching vibrations increased with increasing cell damage.

Changes in the 1800–400 cm^−1^ regions demonstrated that the amide I peak (ca. 1670–1700 cm^−1^) was possibly confounded by the ester C=O stretching of the lipid component of the phospholipid bilayer, since it was also shifted. The shift was recorded as the difference between the average amide I peak position of the treated bacteria and the average amide I peak position of the pristine bacteria (Figure 3). Following an analysis of all the bacteria, the amide I peak was shifted to a higher frequency (by up to almost 22 cm^−1^) as the bactericidal activity of the compound on the bacteria increased. The region between 1650 and 1615 cm^−1^ was a combination of amide and C-N stretching vibrations from R-NH_3_^+^ and R-NH_2_. In all the bacteria, this region showed a reduction in absorbance that made the amide I confounded with the ester C=O peak appear sharper. From an inspection of the spectra, the reduction in absorbance of the 1650 and 1615 cm^−1^ regions became more significant as the cell damage/destruction increased. This variation was quantified by taking the absorbance ratio of the amide I peak confounded with the ester C=O peak to the absorbance at 1620–1618 cm^−1^.

There was also a distinct shift in the peak at ca. 1400 cm^−1^, which may be assigned to the C-H deformation of a methylene group that was directly bonded to C=O or P=O and/or a C–O symmetric stretching of a deprotonated carboxylate group. In the cases of all the bacteria tested, the peak at 1400 cm^−1^ was shifted to a higher frequency (by up to 19 cm^−1^) as the extent of the bacterial cell destruction increased. Other spectral variations included changes in the relative amide I and amide II band intensities and perturbations of the peaks in the region from 1200 to 1000 cm^−1^ that may be assignable to the P–O stretching and C–O and C–O-C bending. The metrics used to monitor the changes in the cell structure on contact with the graphene oxide hybrid antimicrobials are given in Table 1.

The C–O and P–O regions also showed some interesting differences that were associated with changes in the relative levels of C–O and P–O stretching vibrations. Due to the simultaneous changes in relative intensities and shifting, no attempt was made to determine a metric for these bands. Following the treatment of the bacteria, all the antimicrobials resulted in an increase in the relative intensity of the C–O vibrations for all four bacterial species, when compared to the pristine bacteria (the (i) series spectra). For *S. aureus* and *K. pneumoniae* in particular, Ag–GO resulted in a more distinct broadening and shifting of these peaks; this may be indicative of a greater damage inflicted on these organisms by Ag-GO. The exposure of *K. pneumoniae* to the ZnO-GO appeared to have less effect on the relative intensity of the two bands than that of the Ag-GO. The exposure of *E. faecium* to graphite led to a significant reduction in the intensity of the symmetric PO_2_^−^ band; this was accompanied by a blue shift in the asymmetric PO_2_^−^ band. GO exposure had the same effect as graphite but to a lesser extent. With Ag-GO, the effect was less apparent than with GO. ZnO-GO arguably had the least effect on *E. faecium*.

### 2.3. Minimum Bactericidal Concentrations and Peak Changes

**Figure 4:**
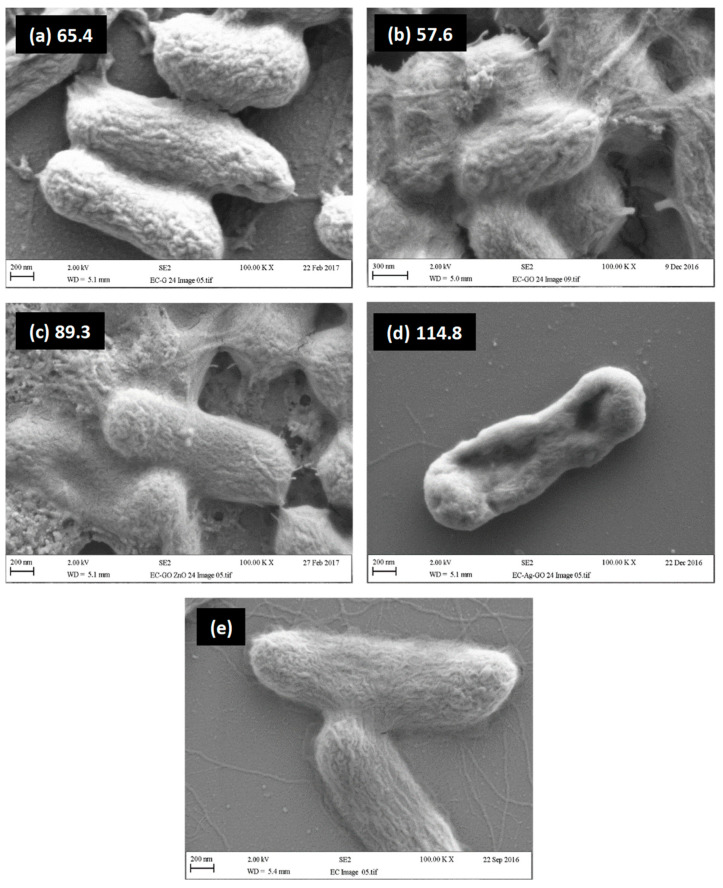
SEM images of *E. coli* after a 24 h exposure to (**a**) graphite (G), (**b**) graphene oxide (GO), (**c**) zinc oxide–graphene oxide hybrid (ZnO-GO), (**d**) silver graphene oxide hybrid (Ag-GO), (**e**) pristine *E. coli*. Note: the PI values are given after the figure legend.

The peak metrics described in Table 1 were correlated with the MBC (42) and the graphene oxide hybrid type is shown in Figure 4. A perturbation index (the sum of metrics one to eight (Table 1) was calculated and used as a single parameter that could be used as an empirical measure of the extent of cell damage inflicted by the graphite and graphene oxide hybrids. It was immediately evident that the Gram-negative bacteria generally showed higher values of the perturbation index at lower MBC values; Ag-GO exposure led to the highest values of the perturbation index and lowest MBC values. It was also interesting to note that the blue shift of the confounded ester and amide I peak was higher for the Gram-negative varieties. This may indicate that the LPS coating of the Gram-negative organisms was more prone to structural disruption than the cross-linked peptidoglycan coating of the Gram-positive bacteria. The latter trends were not so clear for the Gram-positive bacteria; even though a treatment of the bacteria with ZnO-GO gave an MBC of 0.25 mg mL^−1^, and with *S. aureus*, the perturbation index was only 64. Ag-GO also gave an MBC of 0.25 mg mL^−1^ when used against *S. aureus* but had a much higher perturbation index (150). Ag-GO gave an MBC of 0.125 mg mL^−1^ with *E. faecium*, yet the perturbation index was only 90. With GO, *E. faecium* showed a perturbation index of 104 (the highest) but gave an MBC of 0.25 mg mL^−1^. These observations may indicate variations in damage to the membrane; however, the level of damage was probably not severe enough to result in a breaching of the membrane and cell death. In the cases of *S. aureus* and *E. coli*, graphite and GO showed broadly similar performance with some of the metrics for graphite being slightly higher. Against *K. pneumoniae*, the performance of graphite was distinctly poorer than GO. The changes were consistent with an increased disorder in the LPS and peptidoglycan structures that make up the cell walls and confirmed that chemical damage to the bacteria could be related to bactericidal activity.

For the Gram-negative varieties (*E. coli* and *K. pneumoniae*), the value of the perturbation index (the sum of all metric values for the organism in question) increased as the MBC reduced, indicating a correlation with increasing cell damage. The trend was not as strongly evident in the case of the Gram-positive species (*S. aureus* and *E. faecium*). In the Gram-negative organisms, the blue shift of the combined amide I/ester carbonyl peak was greater for each of the candidate antimicrobials. This may indicate that the LPS coating of the Gram-negative organisms was more prone to structural disruption than the cross-linked peptidoglycan coating of the Gram-positive varieties.

### 2.4. Relationship between Visual Assessment of Cell Damage (SEM), FTIR Metrics and MBC Values

A correlation was made on a subjective level to compare the visible cell damage apparent from the SEM images of bacteria exposed to graphite, GO, ZnO-GO and Ag-GO, with the FTIR metrics. The perturbation index (PI) is displayed on the images. Pristine *E. coli* (Figure 4e) appeared to have a rather soft and finely textured cell envelope surface with flagellum evident and when compared to graphite (Figure 4a), the graphitic-treated bacteria looked to be slightly damaged with a possible cell content leakage. The two cells in contact appeared somewhat fused together; this may also have been due to the exudation of retained viscous material. GO and ZnO-GO exposure (Figure 4b,c) resulted in a noticeably greater level of exudation of viscous material and the surface of the cellular membrane looked visibly roughened. This may well have occurred during exposure to the graphene-based materials when in suspension; the exuded material was resistant to the washing sequence used. In addition, the *E. coli* treated with the ZnO-GO demonstrated a distortion of the envelope, and this may have occurred due to a loss of material from within the cell. Exposure to Ag-GO (Figure 4d) appeared to result in a loss of the possibly soft and finely textured material seen on the pristine sample; furthermore, a significant collapse and distortion of the *E. coli* cell envelope had occurred. This was likely due to removal of the surface layer and a loss of material from within the cell followed by the dissolution of the material in the suspension medium and/or removal during the washing sequence. Against *E. coli*, changes to the hydroxyl groups and amide groups on the oligosaccharide repeat units of the O antigen component of the lipopolysaccharide component affixed to the outer phospholipid bilayer and the N-H stretching of the phosphatidyl-ethanolamine component of the phospholipid bilayer with the use of the antimicrobials may explain in part the increased roughness or degradation of the cellular membrane of the bacteria. In addition, significant cell damage was observed following *E. coli* with the Ag-GO, which also gave the highest PI and the joint lowest MBC (with ZnO-GO) of the *E. coli* series.

**Figure 5:**
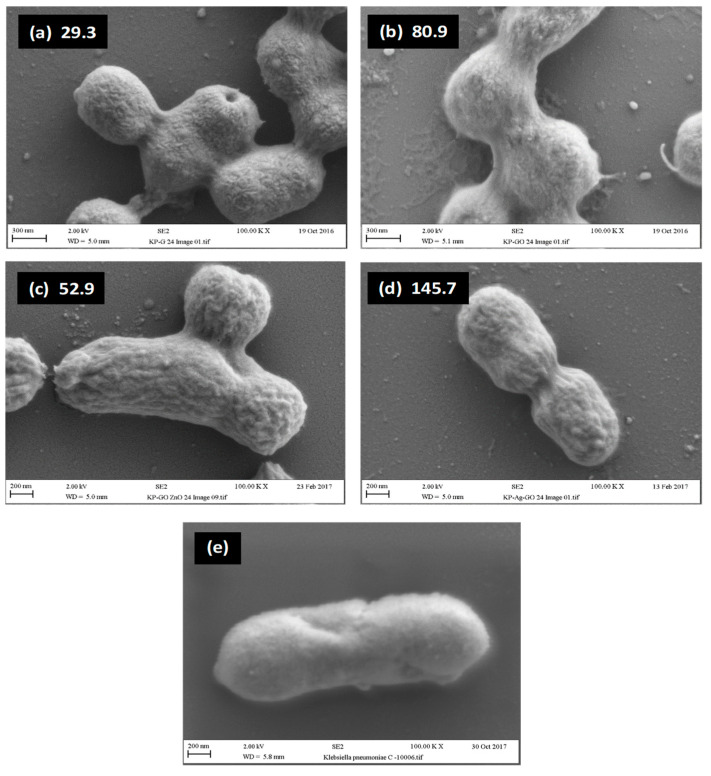
SEM images of *K. pneumoniae* after a 24 h exposure to (**a**) graphite (G), (**b**) graphene oxide (GO), (**c**) zinc oxide–graphene oxide hybrid (ZnO-GO), (**d**) silver graphene oxide hybrid (Ag-GO), (**e**) pristine *K. pneumoniae*. Note: the PI values are given after the figure legend.

Untreated *K. pneumoniae* (Figure 5e) showed a somewhat smoother membrane coating than *E. coli*. Exposure to all the carbon-based materials (Figure 5a–d) led to a roughening of the cell membrane. Following treatment with graphite (Figure 5a) an exudation of viscous material was observed. Even though Ag-GO gave by far the highest PI (and lowest MBC) value of the *K. pneumoniae* series, there was no visual difference observed in the *K. pneumoniae* cellular morphology relative to the bacteria treated with other compounds. Hence, a visual correlation with chemical changes observed by FTIR obviously could not be determined. However, the treatment of *K. pneumoniae* with Ag-GO resulted in a notable increase in bands indicative of changes in the order of LPS or peptidoglycan structures (OH/NH peak width ∆PW(OH + NH); a methyl-asymmetric C-H stretch shift ∆ν_as_(H-CH_2_-); a methylene-asymmetric C-H stretch shift ∆ν_as_(H-CH<); a methyl-symmetric C-H stretch shift ∆ν_s_(H-CH_2_-) and the interaction of the amine groups with candidate antimicrobials (amide I + C=O) to (C-N from R-NH_2_ and R-NH_3_^+^) Abs_C=O_/Abs_C-N_, an alkenic C-H stretch shift ∆ν(H-CH=) suggesting interactions with amides; and in an increase in the relative intensity of C-O vibrations. For *K. pneumoniae*, since Ag-GO resulted in changes and shifting of these peaks, this may be indicative of a greater damage inflicted on these organisms by the Ag-GO.

**Figure 6:**
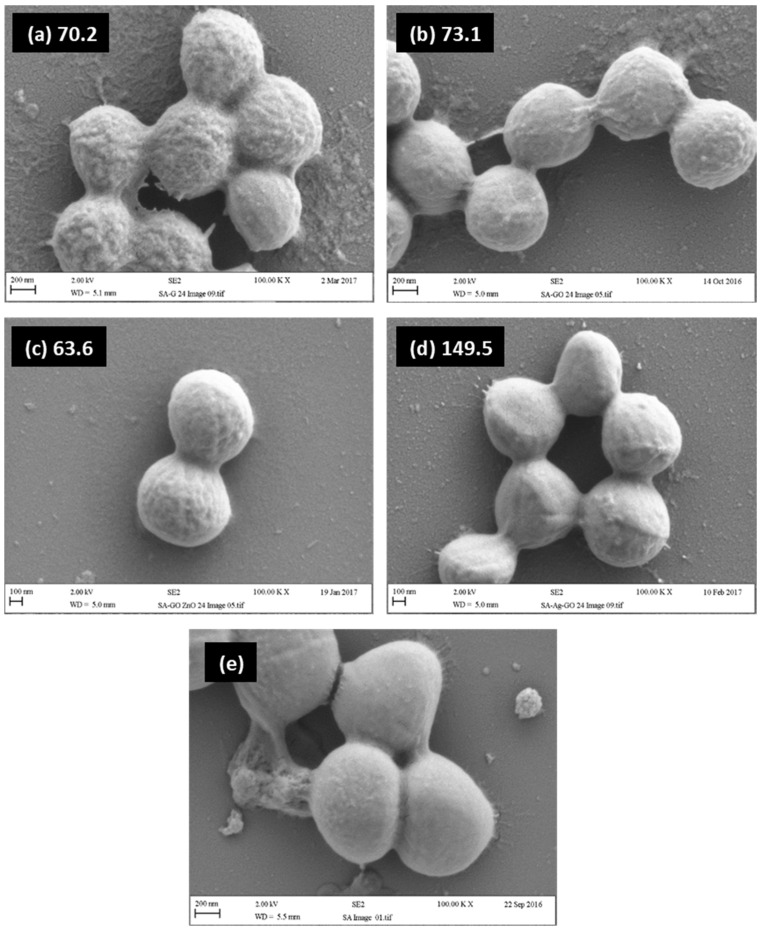
SEM images of *S. aureus* after a 24 h exposure to (**a**) graphite (G), (**b**) graphene oxide (GO), (**c**) zinc oxide–graphene oxide hybrid (ZnO-GO), (**d**) silver graphene oxide hybrid (Ag-GO), (**e**) pristine *S. aureus*. Note: the PI values are given after the figure legend.

The control *S. aureus* (Figure 6e) demonstrated a smooth, regular surface morphology. Exposure to all the carbon-based particles resulted in all the bacterial cell walls becoming roughened (Figure 6a–d), indicating a possible loss of the outer layer and/or the exudation of the retained viscous material. This was particularly true for the bacteria treated with graphite (Figure 6a) which also demonstrated a high amount of exuded material, whilst *S. aureus* treated with GO looked to have exuded some cellular material (Figure 6b). No obvious correlation between visually assessed levels of damage (i.e., amount of exudation, extent of loss of groove definition) and PI could therefore be made. However, against *S. aureus*, a similar pattern of changes in molecular bonding was observed as when the antimicrobials were used against *K. pneumoniae*.

**Figure 7:**
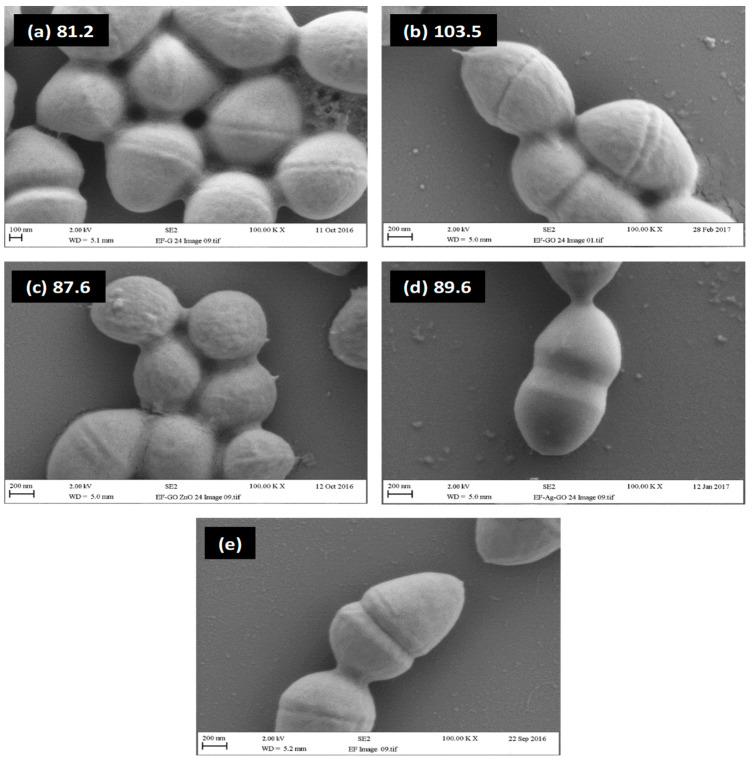
SEM images of *E. faecium* after a 24 h exposure to (**a**) graphite (G), (**b**) graphene oxide (GO), (**c**) zinc oxide–graphene oxide hybrid (ZnO-GO), (**d**) silver graphene oxide hybrid (Ag-GO), (**e**) pristine *E. faecium*. Note: the PI values are given after the figure legend.

Pristine *E. faecium* (Figure 7e) displayed a relatively smooth envelope surface with some coalescence to an adjacent cell. The cells had an acornlike shape with a well-defined groove delineating the acorn cup from the acorn itself. Graphite (Figure 7a) and graphene oxide (Figure 7b) exposure resulted in the conversion of the acorn shape to a more spheroidal shape. Treatment with graphite (Figure 7a) was accompanied by an exudation of cellular contents. Treatment with graphite (Figure 7a) and GO (Figure 7b) and ZnO-GO (Figure 7c) was also accompanied by the exudation of retained viscous material that led to a significant bridging/coalescence of the cells. ZnO-GO exposure resulted in a more significant conversion to a spheroidal shape and a more noticeable shrinkage, the level of exudation and bridging was similar to that observed on exposure to graphite and graphene oxide (Figure 7c). Interestingly, exposure to Ag-GO resulted in a smoothing of the surface texture, a widening of the groove and a transition to a more symmetric elliptical shape (Figure 7d). Furthermore, the level of exudate bridging between cells was also less significant than with the pristine *E. faecium* (Figure 4e). The latter observations were akin to those of Ag-GO/*E. coli* and may reflect similar damage. There was no correlation between the latter and PI as graphene oxide, not Ag-GO, gave the highest PI. However, it was notable that the PI values obtained for *E. faecium* encompassed a narrower range than those obtained for the other organisms, with notably higher values being obtained for graphite and graphene oxide, whilst the PI value obtained from Ag-GO was somewhat lower than that recorded for the other organisms. Ag-GO exposure led to the lowest MBC value, indicating that for *E. faecium*, there was no correlation between visually observed cell damage, PI and MBC.

The exposure of *E. faecium* to the antimicrobial agents also resulted in changes that might be indicative of changes in the LPS or peptidoglycan of the cellular walls (an increased hydrogen-bonded OH/NH peak width ∆PW(OH + NH); a methyl-asymmetric C-H stretch shift ∆ν_as_(H-CH_2_-) and increases in the alkenic C-H stretch shift ∆ν(H-CH=)) suggesting interactions with amides. In addition to an increase in the relative intensity of C-O vibrations against all the antimicrobials, the exposure of *E. faecium* to graphite led to a significant reduction in the intensity of the symmetric PO_2_^−^ band, which was accompanied by a blue shift in the asymmetric PO_2_^−^ band. GO exposure had the same effect as that of graphite but to a lesser extent. With the Ag-GO treatment, the effect was less apparent than with GO.

## 3. Discussion

Near-identical diffuse infrared Fourier transform spectroscopy (DRIFTS) spectra of the Gram-negative *E. coli* and *K. pneumoniae* and a similar spectrum of Gram-positive *S. aureus* was determined. Under the sampling conditions used, it is unlikely that DRIFTS had the same spectral surface bias as when analysing samples diluted in KBr. It may be that a significant portion of the IR radiation penetrated through the layer of cells and reflected off the silicon substrate, thereby giving a substantial double-pass transmission contribution to the spectral data obtained. Therefore, the interior structure of the bacteria was analysed as well as the membrane structure. The latter therefore is likely to prevent, or at least hinder, the differentiation of the membrane characteristics of Gram-positive and Gram-negative bacteria.

In this study, the metal complexes of interest were Ag and Zinc Oxide (ZnO), both of which have been extensively studied to understand their mechanism of action. The most common proposal for silver toxicity is the binding of Ag^+^ ions to polymers and proteins in the bacterial cell wall causing permeability and eventual lysis [42,43], which is supported by morphological evidence of severely disrupted bacterial membranes [44]. However, this still is contentious since it is difficult to determine that this damage is not a by-product of cell death. There is also other evidence suggesting silver can chelate phosphorus-containing groups in deoxyribonucleic acid (DNA), impairing the replication of the organism [45] and disrupting the action of the bacterial electron transport chain [46]. The same issues arise with ZnO and uncertainties surrounding whether the mechanism of action for toxicity is related to the ROS production of the membrane perturbation [47]. There is some evidence of ROS generation with the upregulation of stress response genes katA, ahpC and dnaK in response to treatment of *Campylobacter jejuni* with ZnO nanoparticles [48]. However, despite a dose-dependent increase of ROS species in relation to ZnO being shown in *Pseudomonas aeruginosa*, there was minimal bactericidal activity. An alternative suggestion is that ZnO damaged or disrupted the bacterial membrane, with bacterial viability of *E. coli* and *S. aureus* reduced with the decreasing size of ZnO nanoparticles [49]. Irregularities in the bacterial membranes were identified and the reducing particle size suggested the damage was caused by physical abrasiveness and an increased surface area [50].

A proposed mechanism of action of why graphite was antimicrobial is that it can cause membrane disruption by an oxidation of components of the cell membrane when in direct contact [24]. GO is a nanomaterial made by the oxidation and exfoliation of graphene and is made up of a single layer of hexagonally arranged carbon atoms with oxygen-containing functional groups such as carboxylic acid, ester, ketone, phenols, epoxy, etc. The latter groups are mainly at the platelet edges; however, if the level of oxidation is excessive, a disruption of basal planes can also occur, resulting in quinone and epoxy structures on the platelet surface. This functionalisation creates a hydrophilic compound with favourable surface properties and a contact-based antimicrobial activity against clinically relevant microorganisms [51,52]. The putative mechanisms of the antimicrobial action of GO involve physical and chemical interactions of direct contact between bacteria and the material and/or mediated by lipid peroxidation, both of which can cause membrane damage [25]. The GO material may simply cause membrane damage by sharp edges that can penetrate and disrupt the integrity of the membrane [53] or by lipid peroxidation which is caused by the oxidised state of GO and conjugated/aromatic platelet structures that can stabilise free radicals [54]. It may also be envisaged that the conjugated/aromatic structures of GO and graphite can interact with and stabilise reactive oxygen species, leading to a greater deployment/efficacy of such species. Modes of action may be related to the oxygen-containing functional groups at the platelet edges of GO and the free-radical-stabilising effect of the conjugated/aromatic structure of the platelets (of GO and graphite). The latter in particular may be significant, as reactive oxygen species could be stabilised via interaction with such structures and hence be more prolific and more effective at oxidising the cell membranes. The sharp edges of GO, and indeed graphite (more specifically, the sharp edges of individual graphene layers that comprise graphite), may also contribute mechanical contact damage to the cell walls which may amplify the previously described effects. A combination of all the aforementioned effects with the documented effects of silver enhanced antimicrobial efficacy in a synergistic manner. The activity of GO has been shown to be directly correlated to the surface area and flake size, with a fourfold increase in antimicrobial activity from a flake size of 0.65 to 0.01 μm^2^ [52], indicating it is related to contact. While the effect on bacteria is known for both of these carbon-based products, the precise mechanism of actions is still unclear. With the putative physical properties of graphite and GO and the biochemical properties of Ag and Zn, an antimicrobial surface or biocide with multiple mechanisms of action can be created which is highly effective against clinically relevant organisms. It is of high importance that the mechanisms of action of these metals and emerging antimicrobial compounds are confirmed in order to facilitate the design of these synergistic antimicrobial compounds.

The inner and outer core sugars’ derived units are substituted with phosphate, pyrophosphate, 2-aminoethylphosphate and 2-aminoethylpyrophosphate [27]. It is significant that these charged substituents were closely associated with the Ca^2+^ and Mg^2+^ ions that are key to both membrane structure and function [55]. It may be envisaged that these phosphorous-containing moieties could also interact strongly with particulate antimicrobials and any soluble metal ions that could exude from, for example, graphene oxide metal/metal oxide hybrids.

The phospholipid bilayer is based on fatty acid diesters of phosphatidyl-ethanolamine; the alkyl chains of the fatty acid self-assemble into a bilayer with the phosphatidyl-ethanolamine-derived head groups forming the top and bottom surfaces of the bilayer [56]. The third layer is situated within the periplasmic space, between the inner and outer phospholipid bilayers, consists of peptidoglycan (PG) polymer chains grafted with pentapeptide stems, and the latter are bridged via a range of moieties (depending on the bacterial type) [57]. The molecular architecture of the peptidoglycan layer has been the subject of some debate, but it is agreed that it is a key structural element of the cell due to varying levels of cross-linking [58]. The final (inner) layer is a phospholipid bilayer with no LPS interdigitation. Both the inner and outer phospholipid bilayer walls can be punctuated by inclusions such as protein globules, porins, transmembrane appendages, secretion systems and efflux pumps [56,59,60,61].

With regards to the Gram-positive bacteria, the lengths of both the PG chain and the peptide grafts together with the length and nature of the bridging units have a significant influence on the morphology and properties of the outer layer [58]. The inner layer is a phospholipid bilayer. Due to the differences in the bridging structures of the pentapeptide grafts and differences in the PG chain lengths, the overall cross-link densities and pore sizes of these bacteria differ significantly [28]. The structures of the pentapeptide grafts in *E. faecium* and *S. aureus* are identical but the structures of the cross-links themselves are very different [62]. In *E. faecium*, a single D-aspartate moiety bridges the pentapeptide grafts whereas five glycine repeat units form the pentapeptide bridge them in *S. aureus* [63]. The long bridges between the pentapeptides in *S. aureus* allows a high cross-link density that compensates for the relatively short PG chains, and the pore size is also smaller due to the high cross-link density [64]. As the D-aspartate bridges between pentapeptide chains in *E. faecium* are short, high overall cross-link densities are not possible due to steric restriction, this also leads to larger pore sizes [58]. However, the longer PG chains in *E. faecium* may cancel out the lower cross-link density of the PG, thus possibly cancelling out any detrimental effect on the mechanical properties associated with a lower cross-link density.

In all four bacterial species, blue shifts of the amide I and the α-methylene C-H bend bands were rather difficult to explain as similar observations have not been found in the literature. However, at a fundamental level, a blue shift in a bond vibration frequency can arise from a loss/modification of intermolecular interaction. The hydrogen-bonding interactions of carbonyl groups can cause the bond to lengthen, resulting in a reduced vibration frequency. A reduction in the strength of the hydrogen-bonding interaction will therefore cause a blue shift relative to when the bond was involved in the interaction. A broadening of the NH/OH stretching bands was reported in the literature [65], where it was argued that the effect was related to changes in the protein expression behaviour—the latter was supported by proteomic data. It is also reasonable to consider that the broadening effect could also be due to the disruption of ordered structures leading to a greater variety of hydrogen-bonding interactions. Furthermore, in the literature, changes have also been observed in the PO_2_^−^ and C-O bending vibrations [65]. The reduction in the intensity of the C-NH_2_/C-NH_3_^+^ vibration at 1640–1620 cm^−1^ has been observed in the literature [66] and is considered to be associated with the interaction between the amine groups on the phosphatidyl-ethanolamine component of the phospholipid bilayer and the antimicrobial particle surfaces.

The differently shaped OH–NH stretching band envelope and C-NH_2_/C-NH_3_^+^ vibration (at 1640–1620 cm^−1^) of reduced intensity for *E. faecium* indicated significant structural differences compared to the other organisms. The FTIR data for *E. faecium* indicated that it was sensitive to drying and the effect may well be related to the lower cross-link density and larger pores size of the peptidoglycan outer layer. It is significant that the GO-based materials attacked the bacterial cells via a variety of mechanisms. Such multimode action may prevent the bacteria from developing resistance against such antimicrobials. Further development of GO-based antimicrobials may therefore be important in tackling antimicrobial resistance.

## 4. Conclusions

The selected FTIR spectral metrics could be correlated with cell damage/death arising from exposure to GO hybrids. Ag-GO caused the most severe damage to the cell structure. GO tended to lead to intermediate levels of damage. In some cases (i.e., *E. coli*), G (graphite) led to unexpectedly high levels of damage. ZnO-GO led to relatively low levels of damage. The Gram-negative varieties gave a stronger correlation between FTIR metrics (perturbation index in particular) and the MBC. The blue shift of the confounded ester carbonyl and amide I band was stronger for the Gram-negative varieties. The most reproducible and conveniently used metrics were the hydrogen-bonded OH-NH peak width, the absorbance ratio of the amide I and ester carbonyl to the C-NH_2_/C-NH_3_^+^ stretching bands and the shift in the α-methylene C-H deformation band. A correlation between the extent of visually observed damage and FTIR metrics suggested that the FTIR metrics tended to provide a better assessment of cell damage.

## 5. Material and Methods

### 5.1. Stock Cultures of Bacteria

For the antimicrobial assays, stock cultures of *S. aureus* strain NCTC 4137, *K. pneumoniae* strain NCTC 9633 or *E. coli* strain NCTC 10418 were inoculated onto nutrient agar (NA) or nutrient broth (NB) and incubated at 37 °C for 24 h. Stock cultures of *E. faecium* strain NCTC 7171 were cultured onto Columbia blood agar with 5% horse blood, brain heart infusion agar (BHIA) (Oxoid, Basingstoke, UK) or brain heart infusion broth (BIHB) and incubated in 5% CO_2_ for 24 h at 37 °C. All media were obtained from Oxoid (UK).

### 5.2. Preparation of Microbiological Culture

Microbiological media were inoculated with a single colony of bacteria and incubated overnight in accordance with the conditions in culture and media subsection. Cells were harvested by centrifugation (567× *g* for 10 min) and then washed with 10 mL sterile distilled water and vortexed to ensure an even distribution of the cell suspension. The washed cells were again re-harvested. The pellet was resuspended in 10 mL of broth and vortexed, and the resultant cell suspension was adjusted to an optical density at 540 nanometres (nm) (OD540 nm) of 1.0 using a spectrophotometer. The cell concentrations corresponded to: *E. coli* 4.20 × 10^8^, *S. aureus* 1.30 × 10^8^, *E. faecium* 3.95 × 10^8^ and *K. pneumoniae* 2.82 × 10^8^ colony-forming units per mL (CFU mL^−1^).

### 5.3. Synthesis of Compounds and Characterisation

The synthesis of compounds and characterisation was adapted from Whitehead et al., 2017 [67]. For the synthesis of the compounds, all chemicals (analytical grade or higher) were used as received from Sigma-Aldrich (Gillingham, UK) without any further purification and all solutions were prepared with deionised water of resistivity not less than 18.2 MΩ cm^−1^. Synthetic graphite powder was commercially obtained from Gwent Group (Pontypool, UK).

Graphene oxide (GO) was synthesized by the Hummers method via the oxidation of synthetic graphite [68]. Graphite flakes (5 g) and NaNO_3_ (2.5 g) were combined in 115 mL of concentrated H_2_SO_4_ and stirred for 30 min. Whilst kept in an ice bath (<5 ℃), KMnO_4_ (15.0 g) was gradually added to the suspension and the rate of addition was controlled to keep the reaction temperature below 15 °C. The mixture was heated to 35 °C for 30 min and underwent continuous stirring producing a brown paste. A further dilution was made by adding 250 mL of water to the mixture, and the temperature was increased to 70 °C for 15 min. The resultant mixture was diluted by adding H_2_O until a final volume of 1 L was obtained. Finally, the solution was treated with 15 mL of H_2_O_2_ (30% *w*/*w*) to terminate the reaction, at which stage the solution became yellow in appearance. For purification, the mixture was filtrated and the obtained solid was washed thoroughly with Milli Q water several times in order to avoid sulphate contamination. After purification, the powder was dried at 60 °C for 48 h. In the preparation of the Ag-GO, a sonochemical reduction method was utilised [69]. Following the preparation of the GO, 0.5 g was added to 150 mL of ethylene glycol and sonicated for 30 min. In a separate vesicle, 1.0 g of silver nitrate was added to 20 mL of ethylene glycol and sonicated for 30 min. The silver nitrate dispersion was added dropwise to the GO solution whilst undergoing sonication for 30 min to produce a homogeneous mixture. Finally, 50 mL of 0.1 M NaBH_4_ was added to the resultant Ag-GO mixture, and a further 30 min of sonication was performed. The product was purified with repeated steps of H_2_O and ethanol washing, after which the solution was dried at 50 °C.

The ZnO-GO was fabricated by dissolving 5.0 g GO in 200 mL of *N*,*N*,-dimethylformamide (DMF), along with 20 mL of 1M zinc acetate dihydrate (pH of 6.5). The homogeneous solution was heated to 60 °C and was stirred continuously for 120 min, after which the solution was heated to 250 °C. Following solvent evaporation, partial ZnO/ZnOHGO was produced. The resulting dried product was collected and ground in an agate mortar prior to being annealed at 450 °C for 120 min within atmospheric conditions to obtain the final ZnO-GO product [70].

### 5.4. Preparation of Silicon Wafers

Bacterial suspensions were grown for 24 h in their respective conditions with different compounds and 10 µL of suspension was pipetted onto 1 cm × 1 cm coupons cut from silicon wafers (Montco Technologies, Spring City, PA, USA) before being dried in a class-two laminar air flow cabinet for 1 h. After drying the substrata with the cells, the coupons were immersed in 4% glutaraldehyde overnight at 4 °C. The samples were thoroughly rinsed with 10 mL of distilled water, using a distilled water bottle at a 45° angle with a 3 mm nozzle. They were dried again for 1 h in a class 2 flow hood, then they were immersed for 10 min in different concentrations of ethanol (30%, 50%, 70%, 90% and 100%) and dried for 1 h in the class 2 flow hood. The latter samples were used for the FTIR analysis using a diffuse reflectance cell. Another set of samples were used for SEM imaging and were attached to SEM stubs with carbon tabs prior to being sputter-coated with a gold and palladium coating (model: SC7640, Polaron, Au/Pd target, deposition time: 1.5 min). SEM was carried out using a Supra 40VP with SmartSEM software v5.6 (Carl Zeiss Ltd., Cambridge, UK). All images were captured at a 15,000× magnification. This protocol was adapted from Rajab et al., 2017 [71].

### 5.5. Minimal Bactericidal Concentrations (MBC)

The method for MBC was adapted from Vaidya et al., 2017 [21]. One millilitre of Triphenyl tetrazolium chloride (TTC) blue metabolic dye (Sigma-Aldrich, UK), was added into 9 mL of the OD-adjusted cell suspension so that the working concentration of the dye was 0.15% *w*/*v*. To determine the MIC, 100 μL of the test samples and acid controls were added to a 96-well flat-bottomed microtiter plate (MTP). One hundred microliters of bacterial suspension with TTC was then added; the first column of the cell/metal ion suspension was mixed, then 100 μL of the sample/bacterial mix was transferred to column 2, and repeated until column 10. To column 11, 100 μL of bacterial suspension without a metal (positive control) was added, and to column 12, 100 μL of sterile broth was added (negative control). After incubation, the minimal inhibitory concentration (MIC) was recorded as the lowest concentration that inhibited the visible growth of the bacteria. Growth was indicated by a change of colour in the well to dark blue/purple. Twenty-five microliters of culture was taken from the first well that showed no growth and the last well that demonstrated growth and was pipetted onto agar plates using Miles and Misra’s methodology [72]. After incubation for 24 h, the lowest-concentration well sample that showed no bacterial growth on the agar plate was determined to be the MBC for that test sample (*n* = 3).

### 5.6. Fourier Transform Infrared Spectroscopy

The dried suspensions of bacterial cells on 1 cm × 1 cm coupons cut from silicon wafers were analysed in a Spectra-Tech diffuse reflectance cell, fitted in a Thermo-Nicolet Nexus FTIR spectrometer bench. The type of diffuse reflectance cell used had two hemispherical mirrors that could be parted to reveal the sample holder. The coupons were placed on the alignment mirror and the sample holder moved down to obtain the maximum IR throughput. Spectra were made up of 164 coadded scans with the resolution set to 4 cm^−1^. The diameter of the area occupied by the dried bacterial cell suspensions (the suspension dot) was between 5 and 6 mm. The diameter of the IR beam was 1.5–2 mm. The background was taken using the aluminium alignment mirror. As no first-order differential peaks were observed in the spectra, a Kramers–Kronig correction was not required, thereby indicating that the spectra were made up of mainly diffuse reflected components. Some peaks assignable to the silicon wafer substrate were apparent at the long wavelength end of the spectrum and were removed by subtraction, using a spectrum of a pristine silicon wafer as reference. The spectral metrics in Table 1 were performed on baseline corrected spectra. Values of a given metric were obtained from each of the four replicate spectra and the average value, together with the standard deviation reported; the error bars on graphs are +/− the standard deviation values obtained. In cases where difference values were calculated (i.e., peak shifts), the standard deviation for each value was added in quadrature. Dried suspensions were obtained after a 0 h and 24 h exposure to the candidate antimicrobials (*n* = 4). Only data from the 24 h exposure were reported as it enabled a comparison with MIC, MBC data and SEM images after exposure to the various antimicrobial agents.

## Figures and Tables

**Table 1:** FTIR peak metrics selected for the monitoring of bacterial cell damage.

Metric Number	Metric	Abbreviation	Wave Number Range (cm^−1^)	Comments
1	Amide 1 and ester shift	∆ν(C=O)_Amide1+ester_	1700–1680	Interaction with amide I
2	Absorbance ratio (amide I + C=O) to (C-N from R-NH_2_ and R-NH_3_^+^)	Abs_C=O_/Abs_C-N_	1670–1615	Interaction of amine groups with candidate antimicrobials
3	a-methylene and/or C-O (deprotonated carboxylate) shift	∆(δα > CH-H) + ν_s_(C-O)	1400–1430	Indicative of changes in the order of LPS or peptidoglycan structures
4	Change in hydrogen bonded OH/NH peak width	∆PW(OH + NH)	3600–2800	Indicative of changes in the order of LPS or peptidoglycan structures
5	Alkenic C-H stretch shift	∆ν(H-CH=)	3060–3090	Interaction with amides
6	Methyl asymmetric C-H stretch shift	∆ν_as_(H-CH_2_-)	2955–2980	Indicative of changes in the order of LPS or peptidoglycan structures
7	Methylene asymmetric C-H stretch shift	∆ν_as_(H-CH<)	2930–2942	Indicative of changes in the order of LPS or peptidoglycan structures
8	Methyl symmetric C-H stretch shift	∆n_s_(H-CH_2_-)	2873–2883	Indicative of changes in the order of LPS or peptidoglycan structures
9	Perturbation index	PI	Sum of metrics 1 to 8 *	Provides a single figure indicating the amount of spectral perturbation that may be related to cell damage

* Note that in order to get reasonable figures for a graphical representation, ∆PW(OH + NH) was divided by 10 and Abs_C=O_/Abs_C-N_ was multiplied by 10 before combining with the other metrics to obtain the perturbation index (PI).

## Data Availability

Data will be available on reasonable request.
